# Human cytomegalovirus and Epstein–Barr virus infections occurring early after transplantation are risk factors for antibody-mediated rejection in heart transplant recipients

**DOI:** 10.3389/fimmu.2023.1171197

**Published:** 2023-05-15

**Authors:** Alda Saldan, Carlo Mengoli, Dino Sgarabotto, Marny Fedrigo, Annalisa Angelini, Giuseppe Feltrin, Antonio Gambino, Gino Gerosa, Luisa Barzon, Davide Abate

**Affiliations:** ^1^ Department of Molecular Medicine, University of Padova, Padova, Italy; ^2^ Transplant Infectious Disease Unit, Padova General Hospital, Padova, Italy; ^3^ Department of Cardiothoracic and Vascular Sciences, University of Padova, Padova, Italy; ^4^ Transplant Coordination Center-Veneto Region, Padova, Italy

**Keywords:** human cytomegalovirus, Epstein Barr virus, heart transplantation, antibody mediated rejection, viral immunology

## Abstract

**Background:**

Antibody-mediated rejection (AMR) is a serious complication affecting the survival of patients receiving transplantation. Human cytomegalovirus (CMV) and Epstein–Barr virus (EBV) are common viral infections that occur after transplantation, frequently emerging as viral reactivation in donor grafts or transplant recipients. The present study aimed to investigate the association between CMV and EBV infections and early-onset AMR.

**Materials and methods:**

This study was conducted at the Heart Transplantation Center of Padova General Hospital and included a cohort of 47 heart transplant recipients (HTxs), including 24 HTxs diagnosed with AMR and 23 control HTxs with no episodes of AMR. Only early cases of CMV and/or EBV infections (1–90 days after transplantation) were considered. Fisher’s exact test and logistic regression analysis were used to statistically analyze the correlation and association between AMR and CMV or EBV infection.

**Results:**

We observed a positive statistical association between CMV and EBV infections (two-sided Fisher’s exact test, p = 0.0136) and between EBV infection and AMR (two-sided Fisher’s exact test, p = 0.0034). Logistic regression analysis revealed a direct statistical association between CMV and EBV infections and AMR risk (p = 0.037 and 0.006 and odds ratio = 1.72 and 2.19, respectively). AMR occurrence was associated with increased viral loads of both CMV and EBV early after transplantation.

**Discussion:**

These findings suggest the role of CMV and EBV infections as relevant risk factors for AMR in HTxs for the first time.

## Introduction

Heart transplantation is a life-saving surgical procedure that is performed in the final stage of heart disease. Despite tremendous progress in transplant management, several post-transplant conditions, including acute and chronic graft rejection, still represent major complications that affect the functionality of durable and stable grafts (1, 2). In general, T-cell immune responses play a critical role in both acute and chronic rejection; however, antibody-mediated rejection (AMR) is one of the most insidious and abrupt complications possibly arising after transplantation. AMR is one of the most important reasons for the failure of heart transplantation and is associated with a worse prognosis and higher rates of cardiac allograft vasculopathy (CAV), hemodynamic complications, and death (3–11). AMR may develop not only in the early post-transplantation phase but also in the late post-transplant phase and is mediated by the presence of donor-specific anti-HLA antibodies (DSAs) (12–16). DSAs mediate graft tissue damage and rejection by mainly activating the complement system, although a study has also described a complement-independent mechanism (17). AMR ultimately leads to graft and endothelial damage, loss of graft function, and graft rejection (18). Nevertheless, the biological and pathological events that trigger AMR are either not known or only partially defined.

Active cytomegalovirus (CMV) and/or Epstein–Barr virus (EBV) infections are major threats to heart transplant recipients (HTxs) (19–21). Both these infections can occur owing to latent viral reactivation in seropositive donor grafts or transplant recipients. In HTxs, symptomatic CMV and EBV infections can lead to CMV disease, EBV-related post-transplant lymphoproliferative disease, and death (22, 23). CMVs and EBVs are large DNA viruses that modulate host cell gene expression via various piracy and decoy mechanisms (24). Therefore, concomitant EBV and CMV viral replication may synergistically affect the assets and stability of the immune system. Moreover, EBV has a well-defined tropism for B cells, and its genome can persist lifelong after lytic infection. The present study aimed to elucidate the potential role of early-onset active CMV and EBV infections as risk factors for AMR in a case–control cohort comprising 47 HTxs, including 24 HTxs with AMR and 23 controls.

## Materials and methods

### Patients and clinical definitions

This nested case–control study comprised a cohort of 47 HTxs. The study group included 24 patients diagnosed with AMR, and the control group included 23 patients without serologic/bioptic or clinical evidence of AMR. All 47 HTxs were seropositive for both CMV and EBV before transplantation. Patients were enrolled between June 2010 and June 2015 at the Heart Transplantation Center of Padova General Hospital. All transplant procedures and follow-ups were conducted at the Cardiothoracic Surgery Unit of Padova General Hospital. The Internal Review Board of Padova General Hospital approved all the medical procedures (protocol #NRC AOP0401). Patients were enrolled after obtaining informed consent to participate in the study. The participating patients were provided with a written informed consent form along with a letter to their primary care physician indicating the purpose of the study and collection and handling of patient data. Patients with pre-existing or acquired immunodeficiency were not included in the study. All patients with AMR presented *de novo* DSA. The participants in the control group were selected based on their CMV and EBV serostatus (R+) and similarities in the immunosuppressive regimen (**Table 1**). The control group was similar to the AMR group because it comprised participants selected within the same transplant center. Both groups were treated with similar standards of care protocols and procedures.

**Table 1:** Clinical and therapeutic conditions of the patients.

	AMR group		Control group		p-Value
	Absolute number	%	Absolute number	%	
Number of patients	24		23		
Age (median and range)	58 (4–77)		66 (35–75)		NS
Sex					
Male	20	83	23	100	NS
Female	4	17	0	0	NS
Immunosuppressive regimen					
CNI	21	88	23	100	NS
With MMF	11	52	10	43	NS
With AZA	2	10	3	13	NS
With mTOR inhibitors	5	24	2	9	NS
With steroids	5	24	4	17	NS
Acute rejection score (≥2R)	11.5		11		NS
CAV	6	25	7	30	NS
Active CMV infection	10	42	4	17	NS
Active EBV infection	13	54	2	9	0.001

CNI, calcineurin inhibitors; MMF, mycophenolate mofetil; AZA, azathioprine; mTOR, mammalian target of rapamycin; CAV, cardiac allograft vasculopathy; NS, not significant; AMR, antibody-mediated rejection.

### Criteria for AMR diagnosis and treatment

AMR was diagnosed according to the International Society for Heart and Lung Transplantation guidelines (7, 11, 25–31). The patients with AMR who were included in this study were pAMR1i+, pAMR1h+, pAMR2, and pAMR3. Twenty-two patients with asymptomatic AMR cases did not receive any specific treatment, whereas two with symptomatic AMR received plasmapheresis, intravenous immunoglobulin (Ig), and anti-CD20 antibodies. Patients with symptomatic AMR had reduced left ventricular ejection fraction and an abnormal electrocardiographic profile.

### Evaluation of CMV and EBV DNAemia and CMV and EBV serology tests

Routine surveillance for viral reactivation or infection comprised weekly determination of CMV and EBV DNAemia during the first 100 days post-transplantation; this surveillance continued if there were clinical indications for infection. CMV and EBV DNAemia were evaluated by performing real-time polymerase chain reaction (PCR) on the Abi Prism 7900 HT system (Applied Biosystems, Carlsbad, CA, USA) using a system developed in-house (32, 33). The serologies of CMV IgG and IgM were assessed using diagnostic-grade IgG and IgM ELISA kits (Enzygnost, Dade Behring, Marburg, Germany). The EBV serology test was performed to evaluate IgG positivity for viral capsid antigen (VCA), Epstein–Barr nuclear antigen (EBNA) (Novagnost, Siemens, Marburg, Germany), and early antigen (EA) (Virion, Siemens, Marburg, Germany).

All transplant recipients received transplant conditioning therapy via the administration of anti-thymocyte globulin (1 mg·kg^−1^·day^−1^) for 4 days post-transplant. The immunosuppressive maintenance schemes are presented in **Table 2**. Transplant recipients underwent preemptive treatment for CMV and EBV infections once viral loads reached >5,000 copies/ml of whole blood. Interlaboratory quantitative PCR variability was determined as previously described (34). Preemptive treatment for CMV infection included oral administration of valganciclovir (Valcyte; Roche, Basel, Switzerland) at a standard dose (900 mg/BD) or intravenous administration of ganciclovir (5 mg/BD), corrected according to renal function. EBV infection was treated by administering valaciclovir (3,000 mg/BD); in cases of persistent EBV DNAemia, immunosuppressive treatment was tapered. Preemptive antiviral therapy was considered successful when two sequential CMV or EBV DNAemia test results were negative. No cases of CMV-resistant strains were detected among the transplant recipients.

**Table 2:** Fisher’s exact test.

Variable pairs	Test and significance	Correlation
Early EBV/AMR	Two-sided Fisher’s exact test, p = 0.0034	Positive
Early EBV/Early CMV	Two-sided Fisher’s exact test, p = 0.0136	Positive

The two-sided pairwise correlation included the following variables: AMR, pre-transplant CMV/EBV seropositivity, and early (1–90 days after transplantation) infections. AMR is the dependent variable, whereas CMV or EBV infection is the explanatory variable. Only statistically significant associations are reported.

AMR, antibody-mediated rejection; CMV, human cytomegalovirus; EBV, Epstein–Barr virus.

### Statistical analysis

Statistical analysis was performed using Stata 13 software (StataCorp, College Station, TX, USA). The variables considered for statistical analyses were CMV/EBV seropositivity before transplantation and early CMV and/or EBV DNAemia (occurring 1–90 days after transplantation). For Fisher’s exact test, all variables were coded as categorical or binary (yes/no). Logistic analysis was performed to investigate the association between viral infection (predictor variables) and AMR (dependent variables). Statistical significance was set at a p-value of <0.05.

## Results

**Figure 1:**
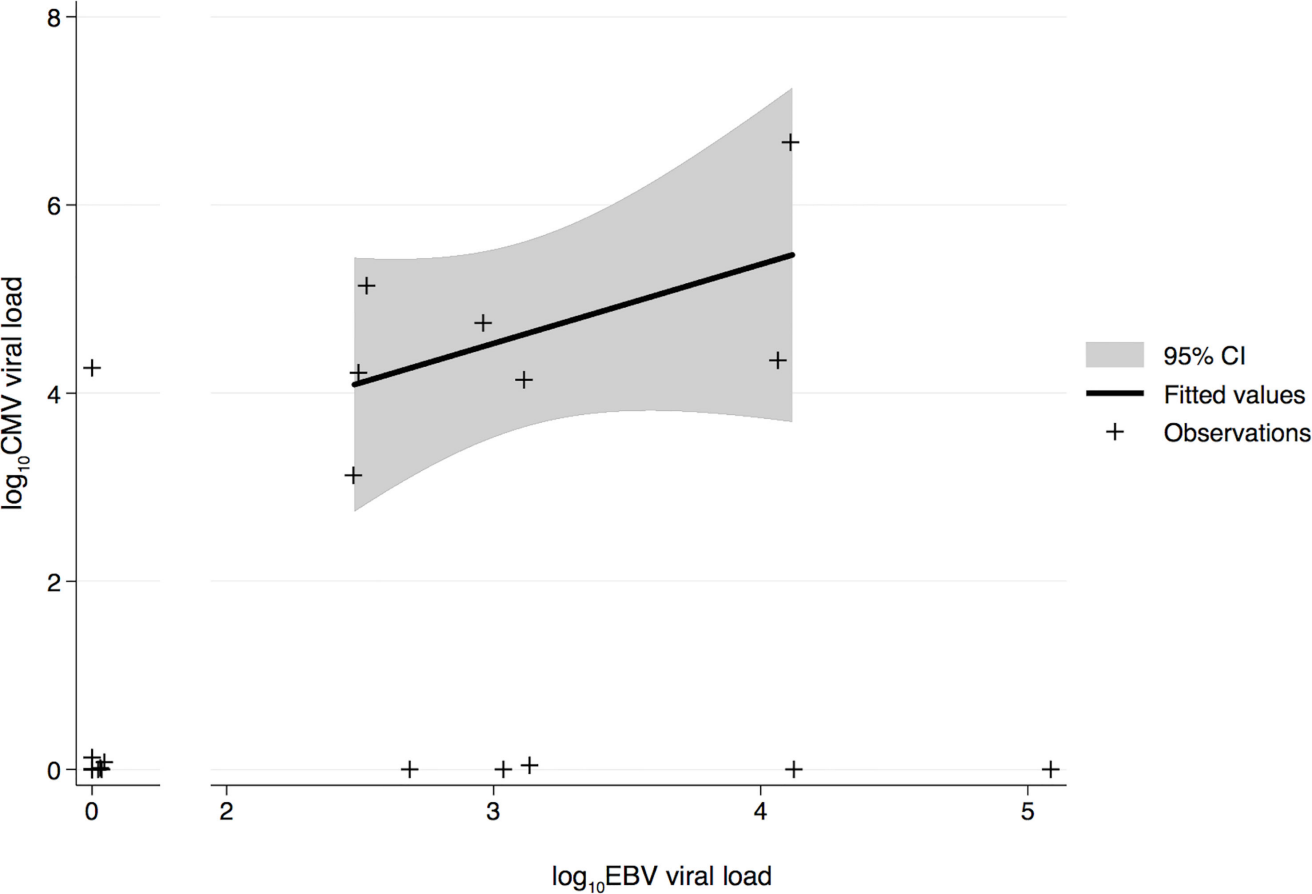
Log-transformed quantitative comparison of CMV and EBV viral load during the early post-transplant phase in transplant recipients with AMR. The solid line indicates the fitted values, gray area indicates the confidence interval, and crosses indicate the data points. CMV, human cytomegalovirus; EBV, Epstein–Barr virus; AMR, antibody-mediated rejection.

The association between CMV and EBV infections and AMR was investigated in 24 HTxs with AMR. The control group comprised 23 adult HTxs without AMR. **Table 1** presents the characteristics of the two groups. As seen in **Table 1**, the prevalence of active EBV infection was significantly higher in the AMR group than in the control group; however, no differences were observed in terms of age, sex, immunosuppression, acute rejection, and CAV. Moreover, neither immunosuppressive reduction nor total lymphocyte count was significantly associated with AMR (data not shown). Comparison of CMV and EBV viral loads (expressed as DNAemia levels) revealed that CMV and EBV infections frequently occurred simultaneously in the AMR group (**Figure 1**) and preceded and/or were concomitant with AMR events. This positive correlation between CMV and EBV infections in patients with AMR was also confirmed using the two-sided Fisher’s exact test (p = 0.0136, **Table 2**). The two-sided Fisher’s exact test also revealed a positive statistical correlation between early-onset EBV infection and AMR occurrence (two-sided Fisher’s exact test, p = 0.0034, **Table 3**).

**Table 3:** Coefficients of the logistic regression model with AMR as the dependent variable and early EBV viremia as the predictor.

AMR	Odds ratio	Standard error	z	p	95% confidence interval	
Early EBV infection (log_10_ viral load)	2.1901	0.6294	2.73	0.006	1.2469	3.8467
Intercept	0.5642	0.2037	−1.58	0.113	0.2780	1.1450

The coefficients are presented in exponential form. EBV is a continuous variable, log_10_ EBV viral load. Log-likelihood = −26.9291. LR chi2(1) = 11.28. Prob chi2 = 0.0008. Pseudo R^2 =^ 0.1731.

AMR, antibody-mediated rejection; EBV, Epstein–Barr virus.

The association between early CMV or EBV infection and AMR was also investigated using logistic regression analysis, with CMV or EBV as predictors of AMR (dependent variable) (**Tables 3**, **4**). Early-onset EBV and CMV infections were significantly associated with AMR (p = 0.006 and 0.037, with odds ratios of 2.19 and 1.72, respectively). CMV PCR and EBV PCR performed on AMR biopsies were positive only in cases of high viral load in the blood.

**Table 4:** Coefficients of the logistic regression model with AMR as the dependent variable and early CMV viremia as the predictor.

AMR	Odds ratio	Standard error	z	p	95% confidence interval	
Early CMV infection (log_10_ viral load)	1.7207	0.4484	2.08	0.037	1.0325	2.8678
Intercept	0.7375	0.2593	−0.87	0.386	0.3703	1.4690

The coefficients are in exponential form. CMV is a continuous variable, log_10_ CMV viral load. Log-likelihood = −25.5624. LR chi2(1) = 7.00. Prob chi2 = 0.0081. Pseudo R^2 =^ 0.1205.

AMR, antibody-mediated rejection; CMV, human cytomegalovirus.

## Conclusions

Active CMV and EBV infections are considered major risk factors for acute T cell-mediated graft rejection, contributing to the accelerated progression of atherosclerosis and allograft loss (35–37). The basic mechanisms have been widely investigated and involve complex immunomodulatory processes that ultimately lead to loss of graft function (38–41). In particular, both CMV and EBV contain large DNA genomes that produce various decoy molecules that act at both the intracellular and extracellular levels and interfere with several stages and critical points of immune regulation (42–45). In the present study, several statistical approaches were used to explore the association between CMV and EBV infections and AMR risk among HTxs. Fisher’s exact test and logistic regression analysis revealed a strong statistically significant association between concomitant CMV and EBV infections and AMR, particularly in the presence of high viral loads. To the best of our knowledge, this strong relationship between EBV and CMV infections and AMR is a novel finding and has not been reported previously. We hypothesize that EBV infection plays a primary role in promoting AMR because EBV productively infects B cells, leading to abnormal B-cell proliferation and aberrant B-cell responses, which may ultimately increase the risk of developing DSAs and AMR in transplant recipients. In this speculative scenario, CMV may be a relevant cofactor because it is well established that CMV infection favors the emergence of other opportunistic infections, including EBV. Furthermore, CMV and EBV may be involved in AMR development by influencing both direct and indirect cellular pathways because AMR biopsies were positive for both CMV and EBV when blood viral loads were high. In the future, it would be interesting to assess whether EBV- and CMV-specific cell-mediated immunity (CMI) plays a role in preventing AMR because, in several transplant settings, virus-specific CMI plays an essential role in controlling viral replication (46–50). Overall, this is the first study to report a statistically significant correlation between CMV/EBV infection and AMR in HTxs.

This study has some limitations. It was a single-center study. Further, a small number of patients were enrolled in the study, and the primary focus was on patients with early post-transplant AMR. Nevertheless, AMR represents a rare event after heart transplantation. Moreover, other infectious agents may contribute to AMR onset; in the patients enrolled in this study, no clinical signs of herpes simplex virus 1/2 or varicella-zoster virus infection were observed. However, investigating the presence of other viral and non-viral infectious agents may provide critical insights into the microbial contribution to AMR initiation. Without a doubt, this finding needs to be further investigated in larger multicenter studies with more patients. We envision that if further studies confirm the association between early and overt EBV and CMV replication and AMR, strategies to prevent CMV and EBV infections using early post-transplant antiviral therapies may help reduce the incidence of AMR and improve the successful outcomes of heart transplantation.

## Data availability statement

The raw data supporting the conclusions of this article will be made available by the authors, without undue reservation.

## Ethics statement

The studies involving human participants were reviewed and approved by NRC AOP0401. The patients/participants provided their written informed consent to participate in this study.

## Author contributions

DA, AS, GF, and CM collected and analyzed the data and wrote the manuscript. DS, GG, AG, and LB supervised the study. CM performed statistical analyses. MF, GF, and AA analyzed the AMR data. All authors contributed to the article and approved the submitted version.

## References

[1] Costello JP Mohanakumar T Nath DS. Mechanisms of chronic cardiac allograft rejection. Tex Heart Inst J (2013) 40(4):395–9. PMC3783121 24082367

[2] Weiss MJ Madsen JC Rosengard BR Allan JS. Mechanisms of chronic rejection in cardiothoracic transplantation. Front Biosci (2008) 13:2980–8. doi: 10.2741/2903 PMC2867599 17981771

[3] Hammond EH Yowell RL Nunoda S Menlove RL Renlund DG Bristow MR. Vascular (Humoral) rejection in heart transplantation: pathologic observations and clinical implications. J Heart Transplant (1989) 8(6):430–43. 2693662

[4] Michaels PJ Espejo ML Kobashigawa J Alejos JC Burch C Takemoto S. Humoral rejection in cardiac transplantation: risk factors, hemodynamic consequences and relationship to transplant coronary artery disease. J Heart Lung Transplant (2003) 22(1):58–69. doi: 10.1016/S1053-2498(02)00472-2 12531414

[5] Rose EA Smith CR Petrossian GA Barr ML Reemtsma K. Humoral immune responses after cardiac transplantation: correlation with fatal rejection and graft atherosclerosis. Surgery (1989) 106(2):203–7. 2669195

[6] Michaels PJ Fishbein MC Colvin RB. Humoral rejection of human organ transplants. Springer Semin Immunopathol (2003) 25(2):119–40. doi: 10.1007/s00281-003-0139-x 12955463

[7] Reed EF Demetris AJ Hammond E Itescu S Kobashigawa JA Reinsmoen NL. Acute antibody-mediated rejection of cardiac transplants. J Heart Lung Transplant (2006) 25(2):153–9. doi: 10.1016/j.healun.2005.09.003 16446213

[8] Schuurman HJ Jambroes G Borleffs JC Slootweg PJ Meyling FH de Gast GC. Acute humoral rejection after heart transplantation. Transplantation (1988) 46(4):603–5. doi: 10.1097/00007890-198810000-00033 3051572

[9] Colvin MM Cook JL Chang P Francis G Hsu DT Kiernan MS. Antibody-mediated rejection in cardiac transplantation: emerging knowledge in diagnosis and management. Circulation (2015) 131(18):1608–39. doi: 10.1161/CIR.0000000000000093 25838326

[10] Kfoury AG Hammond MEH Snow GL Drakos SG Stehlik J Fisher PW. Cardiovascular mortality among heart transplant recipients with asymptomatic antibody-mediated or stable mixed cellular and antibody-mediated rejection. J Heart Lung Transplant (2009) 28(8):781–4. doi: 10.1016/j.healun.2009.04.035 19632573

[11] Kobashigawa J Crespo-Leiro MG Ensminger SM Reichenspurner H Angelini A Berry G. Report from a consensus conference on antibody-mediated rejection in heart transplantation. J Heart Lung Transplant (2011) 30(3):252–69. doi: 10.1016/j.healun.2010.11.003 PMC3829685 21300295

[12] Clerkin KJ Farr MA Restaino SW Zorn E Latif F Vasilescu ER. Donor-specific anti-hla antibodies with antibody-mediated rejection and long-term outcomes following heart transplantation. J Heart Lung Transplant (2017) 36(5):540–5. doi: 10.1016/j.healun.2016.10.016 PMC5654313 27916323

[13] Barten MJ Zuckermann A. The meaning of donor-specific antibodies after heart transplant. Curr Opin Organ Transplant (2019) 24(3):252–8. doi: 10.1097/mot.0000000000000641 31090632

[14] Su JA Baxter-Lowe LA Kantor PF Szmuszkovicz JR Menteer J. The clinical impact of donor-specific antibodies on antibody-mediated rejection and long-term prognosis after heart transplantation. Curr Opin Organ Transplant (2019) 24(3):245–51. doi: 10.1097/mot.0000000000000636 31090631

[15] Sun Q Yang Y. Late and chronic antibody-mediated rejection: main barrier to long term graft survival. Clin Dev Immunol (2013) 2013:859761. doi: 10.1155/2013/859761 24222777 PMC3816029

[16] Clerkin KJ Restaino SW Zorn E Vasilescu ER Marboe CC Mancini DM. The effect of timing and graft dysfunction on survival and cardiac allograft vasculopathy in antibody-mediated rejection. J Heart Lung Transplant (2016) 35(9):1059–66. doi: 10.1016/j.healun.2016.04.007 PMC5662939 27423693

[17] Valenzuela NM McNamara JT Reed EF. Antibody-mediated graft injury: complement-dependent and complement-independent mechanisms. Curr Opin Organ Transplant (2014) 19(1):33–40. doi: 10.1097/mot.0000000000000040 24316758 PMC4080796

[18] Cross AR Glotz D Mooney N. The role of the endothelium during antibody-mediated rejection: from victim to accomplice. Front Immunol (2018) 9:106. doi: 10.3389/fimmu.2018.00106 29434607 PMC5796908

[19] Razonable RR. Epidemiology of cytomegalovirus disease in solid organ and hematopoietic stem cell transplant recipients. Am J Health Syst Pharm (2005) 62(8 Suppl 1):S7–13. doi: 10.1093/ajhp/62.suppl_1.S7 15821266

[20] Razonable RR Paya CV. Herpesvirus infections in transplant recipients: current challenges in the clinical management of cytomegalovirus and Epstein-Barr virus infections. Herpes (2003) 10(3):60–5. 14759337

[21] Fishman JA. Infection in solid-organ transplant recipients. N Engl J Med (2007) 357(25):2601–14. doi: 10.1016/B978-1-4557-4801-3.00313-1 18094380

[22] Allen U Alfieri C Preiksaitis J Humar A Moore D Tapiero B. Epstein-Barr Virus infection in transplant recipients: summary of a workshop on surveillance, prevention and treatment. Can J Infect Dis Med Microbiol (2002) 13(2):89–99. doi: 10.1155/2002/634318 PMC2094856 18159378

[23] Mattila PS Aalto SM Heikkilä L Mattila S Nieminen M Auvinen E. Malignancies after heart transplantation: presence of Epstein-Barr virus and cytomegalovirus. Clin Transplant (2001) 15(5):337–42. doi: 10.1034/j.1399-0012.2001.150506.x 11678960

[24] Mocarski ES Jr. Immunomodulation by cytomegaloviruses: manipulative strategies beyond evasion. Trends Microbiol (2002) 10(7):332–9. doi: 10.1016/S0966-842X(02)02393-4 12110212

[25] Takemoto SK Zeevi A Feng S Colvin RB Jordan S Kobashigawa J. National conference to assess antibody-mediated rejection in solid organ transplantation. Am J Transplant (2004) 4(7):1033–41. doi: 10.1111/j.1600-6143.2004.00500.xAJT500 15196059

[26] Stewart S Winters GL Fishbein MC Tazelaar HD Kobashigawa J Abrams J. Revision of the 1990 working formulation for the standardization of nomenclature in the diagnosis of heart rejection. J Heart Lung Transplant (2005) 24(11):1710–20. doi: 10.1016/j.healun.2005.03.019 16297770

[27] Leone O Veinot JP Angelini A Baandrup UT Basso C Berry G. 2011 Consensus statement on endomyocardial biopsy from the association for European cardiovascular pathology and the society for cardiovascular pathology. Cardiovasc Pathol (2012) 21(4):245–74. doi: 10.1016/j.carpath.2011.10.001S1054-8807(11)00129-3 22137237

[28] Berry GJ Angelini A Burke MM Bruneval P Fishbein MC Hammond E. The ishlt working formulation for pathologic diagnosis of antibody-mediated rejection in heart transplantation: evolution and current status (2005-2011). J Heart Lung Transplant (2011) 30(6):601–11. doi: 10.1016/j.healun.2011.02.015S1053-2498(11)00796-0 21555100

[29] Fedrigo M Gambino A Tona F Torregrossa G Poli F Benazzi E. Can C4d immunostaining on endomyocardial biopsies be considered a prognostic biomarker in heart transplant recipients? Transplantation (2010) 90(7):791–8. doi: 10.1097/TP.0b013e3181efd059 20811321

[30] Fedrigo M Feltrin G Poli F Frigo AC Benazzi E Gambino A. Intravascular macrophages in cardiac allograft biopsies for diagnosis of early and late antibody-mediated rejection. J Heart Lung Transplant (2013) 32(4):404–9. doi: 10.1016/j.healun.2012.12.017 23498161

[31] Berry GJ Burke MM Andersen C Bruneval P Fedrigo M Fishbein MC. The 2013 international society for heart and lung transplantation working formulation for the standardization of nomenclature in the pathologic diagnosis of antibody-mediated rejection in heart transplantation. J Heart Lung Transplant (2013) 32(12):1147–62. doi: 10.1016/j.healun.2013.08.011 24263017

[32] Cesaro S Murrone A Mengoli C Pillon M Biasolo MA Calore E. The real-time polymerase chain reaction-guided modulation of immunosuppression enables the pre-emptive management of Epstein-Barr virus reactivation after allogeneic haematopoietic stem cell transplantation. Br J Haematol (2005) 128(2):224–33. doi: 10.1111/j.1365-2141.2004.05287.x 15638858

[33] Mengoli C Cusinato R Biasolo MA Cesaro S Parolin C Palu G. Assessment of cmv load in solid organ transplant recipients by Pp65 antigenemia and real-time quantitative DNA pcr assay: correlation with Pp67 rna detection. J Med Virol (2004) 74(1):78–84. doi: 10.1002/jmv.20149 15258972

[34] Lilleri D Lazzarotto T Ghisetti V Ravanini P Capobianchi MR Baldanti F. Multicenter quality control study for human cytomegalovirus dnaemia quantification. New Microbiol (2009) 32(3):245–53. 19845106

[35] Zhou YF Leon MB Waclawiw MA Popma JJ Yu ZX Finkel T. Association between prior cytomegalovirus infection and the risk of restenosis after coronary atherectomy. N Engl J Med (1996) 335(9):624–30. doi: 10.1056/NEJM199608293350903 8687516

[36] Weis M Kledal TN Lin KY Panchal SN Gao SZ Valantine HA. Cytomegalovirus infection impairs the nitric oxide synthase pathway: role of asymmetric dimethylarginine in transplant arteriosclerosis. Circulation (2004) 109(4):500–5. doi: 10.1161/01.CIR.0000109692.16004.AF 14732750

[37] Streblow DN Orloff SL Nelson JA. Acceleration of allograft failure by cytomegalovirus. Curr Opin Immunol (2007) 19(5):577–82. doi: 10.1016/j.coi.2007.07.012 PMC3509935 17716883

[38] Potena L Valantine HA. Cytomegalovirus-associated allograft rejection in heart transplant patients. Curr Opin Infect Dis (2007) 20(4):425–31. doi: 10.1097/QCO.0b013e328259c33b 17609604

[39] Tu W Potena L Stepick-Biek P Liu L Dionis KY Luikart H. T-Cell immunity to subclinical cytomegalovirus infection reduces cardiac allograft disease. Circulation (2006) 114(15):1608–15. doi: 10.1161/CIRCULATIONAHA.105.607549 17015794

[40] Streblow DN Dumortier J Moses AV Orloff SL Nelson JA. Mechanisms of cytomegalovirus-accelerated vascular disease: induction of paracrine factors that promote angiogenesis and wound healing. Curr Top Microbiol Immunol (2008) 325:397–415. doi: 10.1007/978-3-540-77349-8_22 18637518 PMC2699259

[41] Mocarski ES Guo H Kaiser WJ. Necroptosis: the Trojan horse in cell autonomous antiviral host defense. Virology (2015) 479-80:160–6. doi: 10.1016/j.virol.2015.03.016 PMC5115625 25819165

[42] Slobedman B Mocarski ES. Mechanisms modulating immune clearance during human cytomegalovirus latency. Proc Natl Acad Sci USA (2012) 109(36):14291–2. doi: 10.1073/pnas.1212245109 PMC3437855 22949568

[43] Albanese M Tagawa T Hammerschmidt W. Strategies of Epstein-Barr virus to evade innate antiviral immunity of its human host. Front Microbiol (2022) 13:955603. doi: 10.3389/fmicb.2022.955603 35935191 PMC9355577

[44] Iizasa H Kim H Kartika AV Kanehiro Y Yoshiyama H. Role of viral and host micrornas in immune regulation of Epstein-Barr virus-associated diseases. Front Immunol (2020) 11:367. doi: 10.3389/fimmu.2020.00367 32194570 PMC7062708

[45] Guo H Kaiser WJ Mocarski ES. Manipulation of apoptosis and necroptosis signaling by herpesviruses. Med Microbiol Immunol (2015) 204(3):439–48. doi: 10.1007/s00430-015-0410-5 PMC4520828 25828583

[46] Abate D Cesaro S Cofano S Fiscon M Saldan A Varotto S. Diagnostic utility of human cytomegalovirus-specific T-cell response monitoring in predicting viremia in pediatric allogeneic stem-cell transplant patients. Transplantation (2012) 93(5):536–42. doi: 10.1097/TP.0b013e31824215db 22314338

[47] Abate D Saldan A Fiscon M Cofano S Paciolla A Furian L. Evaluation of cytomegalovirus (Cmv)-specific T cell immune reconstitution revealed that baseline antiviral immunity, prophylaxis, or preemptive therapy but not antithymocyte globulin treatment contribute to cmv-specific T cell reconstitution in kidney transplant recipients. J Infect Dis (2010) 202(4):585–94. doi: 10.1086/654931 20594105

[48] Abate D Fiscon M Saldan A Cofano S Mengoli C Sgarabotto D. Human cytomegalovirus-specific T-cell immune reconstitution in preemptively treated heart transplant recipients identifies subjects at critical risk for infection. J Clin Microbiol (2012) 50(6):1974–80. doi: 10.1128/JCM.06406-11JCM.06406-11 PMC3372163 22461674

[49] Abate D Saldan A Mengoli C Fiscon M Silvestre C Fallico L. Comparison of cytomegalovirus (Cmv) enzyme-linked immunosorbent spot and cmv quantiferon gamma interferon-releasing assays in assessing risk of cmv infection in kidney transplant recipients. J Clin Microbiol (2013) 51(8):2501–7. doi: 10.1128/JCM.00563-1JCM.00563-13 PMC3719636 23678073

[50] Abate D Saldan A Forner G Tinto D Bianchin A Palu G. Optimization of interferon gamma elispot assay to detect human cytomegalovirus specific T-cell responses in solid organ transplants. J Virol Methods (2014) 196:157–62. doi: 10.1016/j.jviromet.2013.10.036S0166-0934(13)00450-3 24216234

